# Contrasting Patterns of Serologic and Functional Antibody Dynamics to Plasmodium falciparum Antigens in a Kenyan Birth Cohort

**DOI:** 10.1128/CVI.00452-15

**Published:** 2016-02-05

**Authors:** Arlene E. Dent, Indu Malhotra, Xuelie Wang, Denise Babineau, Kee Thai Yeo, Timothy Anderson, Rhonda J. Kimmel, Evelina Angov, David E. Lanar, David Narum, Sheetij Dutta, Jack Richards, James G. Beeson, Brendan S. Crabb, Alan F. Cowman, Toshihiro Horii, Eric Muchiri, Peter L. Mungai, Christopher L. King, James W. Kazura

**Affiliations:** a Center for Global Health and Diseases, Case Western Reserve University, Cleveland, Ohio, USA; b Department of Pediatrics, Rainbow Babies and Children's Hospital, Cleveland, Ohio, USA; c Department of Epidemiology and Biostatistics, Case Western Reserve University, Cleveland, Ohio, USA; d School of Medicine, Case Western Reserve University, Cleveland, Ohio, USA; e Malaria Vaccine Branch, Walter Reed Army Institute of Research, Silver Spring, Maryland, USA; f Laboratory of Malaria Immunology and Vaccinology, National Institutes of Allergy and Infectious Diseases, National Institutes of Health, Rockville, Maryland, USA; g Burnet Institute, Melbourne, Australia; h Department of Microbiology, Monash University, Melbourne, Australia; i Walter and Eliza Hall Institute of Medical Research, Parkville, Australia; j Department of Molecular Protozoology, Research Institute for Microbial Diseases, Osaka University, Suita, Osaka, Japan; k Division of Vector Borne and Neglected Tropical Diseases, Ministry of Public Health and Sanitation, Nairobi, Kenya

## Abstract

IgG antibodies to Plasmodium falciparum are transferred from the maternal to fetal circulation during pregnancy, wane after birth, and are subsequently acquired in response to natural infection. We examined the dynamics of malaria antibody responses of 84 Kenyan infants from birth to 36 months of age by (i) serology, (ii) variant surface antigen (VSA) assay, (iii) growth inhibitory activity (GIA), and (iv) invasion inhibition assays (IIA) specific for merozoite surface protein 1 (MSP1) and sialic acid-dependent invasion pathway. Maternal antibodies in each of these four categories were detected in cord blood and decreased to their lowest level by approximately 6 months of age. Serologic antibodies to 3 preerythrocytic and 10 blood-stage antigens subsequently increased, reaching peak prevalence by 36 months. In contrast, antibodies measured by VSA, GIA, and IIA remained low even up to 36 months. Infants sensitized to P. falciparum in utero, defined by cord blood lymphocyte recall responses to malaria antigens, acquired antimalarial antibodies at the same rate as those who were not sensitized *in utero*, indicating that fetal exposure to malaria antigens did not affect subsequent infant antimalarial responses. Infants with detectable serologic antibodies at 12 months of age had an increased risk of P. falciparum infection during the subsequent 24 months. We conclude that serologic measures of antimalarial antibodies in children 36 months of age or younger represent biomarkers of malaria exposure rather than protection and that functional antibodies develop after 36 months of age in this population.

## INTRODUCTION

Naturally acquired immunity to malaria develops slowly over time in children in areas of malaria endemicity as a consequence of repeated infections (1). Antibodies play a key role in this immunity, as demonstrated by passive antibody transfer from immune adults to children with clinical malaria, resulting in reduction of symptoms and parasitemia (2, 3). Very young infants <6 months old are relatively protected from clinical malaria, a phenomenon thought to be mediated primarily by maternal IgG antibodies transferred to the fetus in the last trimester of pregnancy. High levels of fetal hemoglobin and nutritional factors may also contribute to decreased malaria susceptibility during early infancy (4–6). Maternal IgG antibodies detectable in cord blood progressively decrease, leaving infants older than approximately 4 to 6 months of age vulnerable to Plasmodium falciparum infection and symptomatic malaria. With repeated infections and increasing age, young infants subsequently acquire IgG antibodies directed against many P. falciparum antigens. The exact antigenic targets of these antibodies, their relative rates of development, and how they function to mediate protection from infection and symptomatic malaria are incompletely understood.

Antimalarial IgG antibodies may potentially mediate protection through multiple functions, e.g., blocking sporozoite invasion of hepatocytes and merozoite invasion of erythrocytes, opsonizing merozoites and infected erythrocytes expressing variant surface antigens on their surface for phagocytosis, and fixation and activation of complement on the merozoite surface with resultant parasite lysis. An increasing number of P. falciparum antigens have been identified as relevant to naturally acquired immunity and, thus, are considered potential vaccine targets (7–9). Evaluation of infant antibody responses to P. falciparum has relied mainly on serologic assays, with some studies indicating that such antibodies are associated with protection from infection and symptomatic malaria (10, 11), while others conclude that they are biomarkers of exposure which, when elevated, are associated prospectively with an increased risk of malaria (6, 12–14). Measurements of alternative functional antibody activities, such as the variant surface antigen (VSA) assay, growth inhibitory activity (GIA), and invasion inhibitory assays (IIA), that reflect impaired interaction of merozoite ligands with the erythrocyte surface membrane have been developed (15–20). There have been few studies of VSA antibodies focused on infants in areas of malaria endemicity (21). Antibodies that inhibit the growth of P. falciparum *in vitro* have been used to assess vaccine efficacy in animal models and malaria-naive human volunteers (22–26). P. falciparum GIA has been associated with protection from infection in children in some studies, but this has not been a consistent finding (15, 27, 28).

The objectives of our study were to advance the knowledge on the breadth and dynamics of various infant antimalarial antibody responses and to determine whether specific antigens and functional antibody responses may be prioritized during the development of naturally acquired immunity in early childhood. Infants born in Msambweni, Kenya, from 2006 to 2009 were followed every 6 months from birth to 36 months. Plasma samples from the study participants were examined for the presence and magnitude of serologically determined IgG antibodies directed against multiple preerythrocytic and blood stage antigens over time. In addition, we measured IgG antibodies to VSA expressed by three different P. falciparum laboratory-adapted isolates: 3D7, a widely used reference isolate; BFD06, isolated from an adult traveler returning from Burkina Faso presenting to the hospital with severe malaria (29); and Msam06, isolated from a child presenting with acute uncomplicated malaria at Msambweni District Hospital, Kenya (30). We evaluated GIA with D10 and W2mef parasites and the acquisition of invasion-inhibitory antibodies directed against MSP1-19 (16, 31) and sialic acid-dependent invasion pathways (32).

## MATERIALS AND METHODS

### Study population and ethical approval.

Healthy, pregnant mothers were recruited from antenatal clinics at Msambweni District Hospital, Coast Province, Kenya, from 2006 to 2009, as previously described (33). Malaria endemicity at the time was in transition from moderate transmission in 2007 to low transmission in 2009 (34). Per the Kenya Ministry of Health national policy, women received intermittent preventive treatment for malaria with sulfadoxine-pyrimethamine beginning in the second trimester in addition to iron, folic acid, and bed nets as part of routine care. Full-term healthy neonates were enrolled in the study. Cord blood was collected after delivery, and blood was collected from the infants (by venipuncture) every 6 months until 36 months of age. All infants with data for this study were born to HIV-negative mothers, and all women provided written, informed consent. The study was approved by the institutional review boards at the Kenya Medical Research Institute and University Hospitals Case Medical Center.

### Samples and sample preparation.

Cord blood was collected in heparinized bags from placentas of full-term deliveries (35). Plasma was stored at −20°C. Cord blood mononuclear cells (CBMCs) were isolated using Ficoll-Paque Plus (GE Healthcare, NJ) density gradient centrifugation and cryopreserved in 90% fetal bovine serum plus 10% dimethyl sulfoxide (Sigma-Aldrich, MO) (35). Heparinized blood from infants was centrifuged, and plasma was stored at −20°C.

### *In utero* sensitization to malaria antigens.

Freshly isolated CBMCs were used to evaluate cytokine production in response to known T-cell epitopes within the C-terminal 83-kDa fragment of merozoite surface protein 1 (MSP1), the 42-kDa fragment of recombinant MSP1 (MSP1_42_ FVO and MSP1_42_ 3D7), and PfP0 (a P. falciparum ribosomal phosphoprotein [36]), as previously described (35). A newborn was considered sensitized to malaria antigens *in utero* when one of the following three conditions were met: (i) by gamma interferon (IFN-γ) enzyme-linked immunosorbent spot assay (ELISPOT), there were >4 cytokine-secreting cells/10^6^ CBMCs in response to MSP1 peptides/MSP1_42_/PfP0 and no secreting cells were detected in negative-control wells containing media alone; (ii) by IFN-γ ELISPOT, in cases where cytokine-secreting cells were observed in negative-control wells, the number of spots generated by MSP1-driven CBMCs was 2-fold greater than control wells; (iii) by enzyme-linked immunosorbent assay (ELISA) for IFN-γ, interleukin 2 (IL-2), IL-5, or IL-13, net cytokine production by CBMCs in response to MSP1 peptides/MSP1_42_/PfP0 was at least 2-fold greater than that of negative-control wells (35). If these criteria were not met, the newborn was categorized as not sensitized.

### Diagnosis of infection by blood smear and PCR.

All blood samples were examined for P. falciparum parasites. Thick and thin blood smears were prepared, stained with 5% Giemsa, and examined by light microscopy for P. falciparum-infected erythrocytes. A slide was deemed negative when no parasites were seen after counting microscopic fields containing at least 200 leukocytes. After Ficoll processing of cord blood and infant blood samples, DNA from 200 μl of the erythrocyte pellet was extracted using the QIAamp 96 DNA blood kit (Qiagen, Valencia, CA). The DNA was subjected to a P. falciparum-specific PCR/ligase detection reaction-fluorescence microsphere assay, as previously described (37). P. falciparum infections (*n* = 39) were detected in asymptomatic pregnant women during this time, and extracted DNA was utilized for MSP1-19 haplotype determination (see below).

### Serologic IgG and IgM antibodies to P. falciparum antigens measured by Luminex multiplex assay.

Recombinant antigens tested included the following proteins: liver stage antigen 1 (LSA1) (38); circumsporozoite protein (CSP) (39); cell-traversal protein for ookinetes and sporozoites (PfCelTOS) (40; serine repeat antigen 5 (SERA5; SE50 [41], SE36 [42]); MSP1 42-kDa fragment (MSP1_42_ 3D7 [43], FVO [44], and FUP [45]); erythrocyte binding antigen 140 (EBA 140) (46), EBA175 (47), and EBA181 (48); and apical membrane antigen 1 (AMA1 3D7) (49) and FVO (50). The proteins AMA1, PfCelTOS, and CSP and the MSP1 alleles were all good manufacturing practice (GMP)-quality proteins and therefore had no host cell contamination. We did not find that there was high reactivity of the responses to the other antigens compared with these GMP-quality proteins. These antigens were selected, as previous cohort studies have indicated that antibodies against them have been generally associated with protective immunity, are targets of acquired invasion-inhibitory antibodies, and are vaccine candidates (17, 51–54). Carboxylated microspheres (Luminex, Austin, TX) were coupled to the proteins using the manufacturer's protocol and as described previously (55–57). Antigen-specific IgG was detected by incubating 1,000 beads of each antigen per well with a 1:1,000 plasma dilution in a final volume of 100 μl. Antigen-specific IgM was detected using the same incubation techniques and a 1:100 plasma dilution. Plasma samples from 4 North American malaria-naive adults were used as negative controls for each plate. A pool of Kenyan adult plasma was used as a positive control on all plates to ensure assay performance and minimal plate-to-plate variation. For IgG antibody responses, the mean fluorescence intensity (MFI) of individual Kenyan plasma samples was normalized to the mean MFI of the negative controls to obviate plate to plate variations. A positive value was assigned if the normalized value was >1.5-fold over malaria-naive controls. For IgM, a positive value was designated if the normalized value was >5-fold over negative controls. All positive values were also greater than the mean plus 3 standard deviations (SD) of the value of the individual negative-control plasma samples.

### Growth inhibition assays.

D10 (D10-PfM3′ [16]) and W2mef parasites were utilized in GIAs, as previously described (15). Briefly, ring-stage parasites were synchronized twice by sorbitol lysis (5% d-sorbitol; Sigma, St. Louis, MO) and allowed to mature to late trophozoite/schizont stages. Parasites were cultured at 4% hematocrit in RPMI 1640 supplemented with 25 mg/ml of HEPES, 2 mg/ml of sodium bicarbonate, 0.5% of Albumax II (Gibco, Grand Island, NY), 2.4 mM l-glutamine, 0.08 mg/ml of gentamicin, and 0.2 mM hypoxanthine. Cultures were maintained at 37°C in an atmosphere of 5% CO_2_, 1% O_2_, and 94% N_2_. Purified parasites were adjusted to 0.5% infected erythrocytes with a final 2% hematocrit, a 1:10 plasma dilution (not heat inactivated at 56°C and, thus, containing complement proteins required for activation by classical and alternate pathways, although freezer storage could make complement function suboptimal), and a 100-μl final volume in 96-well flat-bottom microtiter plates. The cultures were incubated for 26 h to allow for schizont rupture and merozoite invasion (monitored by microscopy to ensure full schizont rupture). Twenty-five microliters of resuspended cultures was removed, fixed with 0.25% gluteraldehyde in phosphate-buffered saline (PBS) for 45 min, and placed in 10× SYBR green I (Molecular Probes, Eugene, OR)—400 μl of 1× PBS for >24 h at 4°C to stain parasite DNA. Stained cells were examined with a BD LSR II flow cytometer to collect data from a minimum of 5 × 10^4^ cells. A Becton-Dickinson fluorescence-activated cell sorter (FACS) equipped with Diva 5.01 software was used to collect and FlowJo 8.5.1 was used to analyze cytometry data. The mean parasitemia for duplicate wells was used to determine the percent GIA calculated with the following equation: 100 − (test plasma parasitemia/nonimmune plasma parasitemia × 100). Plasma samples from 4 North Americans who had never been exposed to malaria were pooled as the nonimmune plasma controls.

### Target-specific invasion inhibition assays.

Methods to quantify MSP1-19 IIA and sialic acid-dependent invasion IIA (Sial Dep IIA) were as described previously (16, 32, 58). Briefly, for the MSP1-19-specific IIAs, D10-PfM3′ and an isogenic D10-PcMEGF parasite line in which the Plasmodium chabaudi orthologue replaces the P. falciparum MSP1-19 region were tested in parallel. Greater inhibition of D10-PfM3′ than D10-PcMEGF parasites was interpreted as inhibitory antibodies targeting Pf-MSP1-19. For Sial Dep IIA, the W2mef isolate and the W2mef with genetic deletion of EBA175 (ΔEBA175) isolate were tested in parallel. W2mef invades predominantly via sialic acid-dependent invasion pathways, and W2mefΔEBA175 invades via sialic acid-independent pathways. Greater inhibition of W2mef parental versus W2mefΔEBA175 was interpreted as inhibitory antibodies to sialic acid-dependent invasion (32). For both assays, ring-stage parasites were synchronized twice by sorbitol lysis and allowed to mature to late trophozoite/schizont stages. Parasites were adjusted to 4% hematocrit with 0.5% P. falciparum-infected erythrocytes, and 50-μl aliquots were placed in 96-well, flat-bottom microtiter plates with an equal volume of 1:5 prediluted plasma in culture medium (final plasma dilution, 1:10; final volume, 100 μl). The same batch of prediluted plasma was added to the two parasite lines in the same assays. The cultures were incubated for 26 h to allow for schizont rupture and merozoite invasion. Then, 25 μl of resuspended cultures was removed, fixed with 0.25% gluteraldehyde—PBS for 45 min, and placed in 10× SYBR green I (Molecular Probes, Eugene, OR)—400 μl of 1× PBS for >24 h at 4°C (15, 58). Stained cells were examined with a BD LSR II flow cytometer to collect data from a minimum of 5 × 10^4^ cells using the Becton-Dickinson FACS Diva 5.01. Ring-stage parasitemia was calculated by quantifying singly infected erythrocytes plus multiply infected erythrocytes (quantified as having two intracellular rings) divided by the total erythrocytes according to flow cytometry gating previously described (15, 58). FlowJo 8.5.1 was used to analyze cytometry data. The mean number of ring-stage parasitemia for duplicate wells was calculated, and results were expressed as a percentage of the ring-stage parasitemia of nonimmune control plasma (derived from 4 North Americans who had never been exposed to malaria) in parallel cultures. The percentage change of invasion inhibition antibodies specifically attributable to MSP1-19 antibodies (MSP1-19 IIA) or Sial Dep IIA was calculated by subtracting the percentage of invasion of the parent P. falciparum strain (D10-PfM3′ or W2mef) relative to nonimmune controls from the percent invasion of the mutated P. falciparum strain (D10-PcMEGF or W2mefΔEBA175) relative to nonimmune controls. A positive response was defined as ≥5% inhibition attributable to MSP1-19 IIA or Sial Dep IIA.

### Antibodies to variant surface antigens.

Anti-VSA IgG antibodies were measured by flow cytometry as previously described, with minor modifications (59, 60); these antibodies appear to predominantly target the infected erythrocyte surface antigen P. falciparum erythrocyte membrane protein 1 (PfEMP1) (19). Three P. falciparum isolates were used: (i) 3D7, a widely used reference isolate, and antibodies to this isolate were previously associated with protection to malaria in Kenyan children (19, 61); (ii) BFD06, which was isolated from an adult traveler with acute severe malaria returning from Burkina Faso in 2006 (29); and (iii) Msam 06, which was isolated in 2006 from a child with acute uncomplicated malaria in Msambweni, Kenya, the study site for this cohort (30). P. falciparum isolates from the two acute malaria patients were adapted to *in vitro* culture. Parasites were grown in group O erythrocytes, synchronized, harvested at the late trophozoite stage, and cryopreserved. All plasma samples were processed at the same time for each individual parasite line. Positive-control plasma consisted of pooled plasma samples from 8 malaria-immune Kenyan adults, and negative-control plasma consisted of pooled plasma samples from 4 malaria-naive North Americans. Thawed parasites were adjusted to 0.2% hematocrit. Two microliters of heat-inactivated test plasma was added to each well of a U-bottom 96-well microtiter plate. Then, 38 μl of the adjusted thawed parasites was added to each well (final plasma dilution, 1:20) and incubated for 60 min at room temperature. Between incubation steps, cells were washed three times with PBS and 0.1% casein. Forty microliters of 1:100 diluted polyclonal rabbit anti-human IgG (Dako, Carpinteria, CA) was added and incubated for 30 min at room temperature, followed by 40 μl of 1:100 diluted Alexa-Fluor-647-conjugated donkey anti-rabbit IgG (Molecular Probes, Eugene, OR) with 10× SYBR green I incubated for 30 min at room temperature. Cells were resuspended in 200 μl PBS—0.1% casein and were examined with a BD LSR II flow cytometer. Infected erythrocytes were differentiated from noninfected erythrocytes by SYBR green fluorescence. For quantification of the Alexa-Fluor, the geometric mean fluorescent intensity (GeoMFI) of each population was used. The magnitude of VSA reactivity was calculated as the GeoMFI of infected erythrocytes minus the GeoMFI of noninfected erythrocytes. A positive response was defined as GeoMFI greater than the mean plus 3 SD of the North American negative controls.

### MSP1-19 haplotype detection.

DNA was extracted from 200 μl of venous blood using the QIAamp DNA blood minikit (Qiagen Corp., Valencia, CA). PCR amplification using MSP1-19-specific and P. falciparum small subunit rRNA-specific primers, the ligase detection reaction–fluorescent microsphere assay (LDR-FMA), and haplotype assignment based on allele-specific mean fluorescence intensity were performed as previously described (55, 62). Importantly, if four alleles (Q, E, KNG, and TSR) were detected in a single sample, we conservatively assumed that only two haplotypes were present. Therefore, the maximum number of haplotypes assigned to any infection was two.

### Statistical analysis.

We estimated the probability of positive antibody responses over time using restricted cubic splines, and we used generalized linear mixed models to estimate the probability of anti-malaria antibody responses over time among newborns sensitized and not sensitized to MSP1 *in utero*. A generalized estimation equation regression model was used to estimate the rate of change in the probability of presence of each antibody response over time and to assess if the mean rate of change in the probability of detecting serological antibodies after 12 months of age was different than the mean rate of change in the probability of detecting VSA, GIA, and IIA antibodies after 12 months of age. Cox proportional hazards regression models were fitted to investigate the association between antibody responses at birth or at 12 months of age and the incidence of P. falciparum infection during the follow-up time period.

## RESULTS

### IgG antibody magnitude and prevalence in cord blood.

We report the malarial antibody dynamics of 84 infants who had approximately 4.3 blood samples per participant with a mean follow-up time of 29 months (minimum, 4 months; maximum, 39 months; median, 33 months). The presence and magnitude of cord blood maternal IgG antibodies are shown in Table 1. Serologic antibodies were common, with anti-AMA1 (3D7 and FVO) having the highest prevalence (97.4%) and magnitude, and antibodies to PfCelTOS and SERA5 (SE50) having the lowest prevalence (14.1% to 19.2%) and magnitude. VSA reactive antibodies were moderately prevalent in cord blood (47.4% to 69.2%). GIAs and Sial Dep IIA were very low in cord blood, with virtually no MSP1-19 IIA detected (6.1%).

**TABLE 1:** Magnitude and prevalence of IgG antibodies in cord blood

Assay/antigen tested (P. falciparum isolate)	Sample size (no.)	Mean (SD)/median (IQR^*a*^)	No. (%) positive
Serology (fold increase in MFI over negative controls)			
LSA1	78	24.7 (42.6)/4.7 (1.6–27.8)	59 (75.6)
CSP	78	29.5 (39.7)/11.4 (3.3–40.0)	68 (87.2)
PfCelTOS	78	0.6 (1.4)/0.0 (0.0–0.0)	15 (19.2)
SE50	78	0.6 (2.1)/0.0 (0.0–0.0)	11 (14.1)
SE36	78	3.1 (6.9)/0.0 (0.0–2.6)	34 (43.6)
MSP1_42_ (3D7)	78	9.3 (11.9)/5.8 (0.0–12.0)	58 (74.4)
MSP1_42_ (FVO)	78	6.5 (9.5)/2.3 (0.0–10.6)	46 (59.0)
MSP1_42_ (FUP)	78	12.2 (13.8)/6.8 (2.4–16.6)	68 (87.2)
EBA140	78	8.7 (19.6)/1.7 (0.0–7.3)	41 (52.6)
EBA175	78	9.9 (14.7)/3.7 (0.0–14.5)	49 (62.8)
EBA181	78	9.6 (17.9)/2.7 (0.0–8.6)	51 (65.4)
AMA1 (3D7)	78	81.0 (46.4)/88.1 (48.4–101.6)	76 (97.4)
AMA1 (FVO)	78	96.7 (61.2)/113.9 (39.5–131.2)	76 (97.4)
Variant surface antigen assay (geometric mean MFI)			
BFD 2006	79	40.2 (56.4)/17.0 (11.0–46.0)	42 (53.8)
Msambweni 2006	79	37.4 (40.9)/21.0 (13.0–44.0)	54 (69.2)
3D7	79	24.2 (28.0)/13.5 (8.0–30.0)	37 (47.4)
Growth and invasion inhibition assay (percent inhibition)			
Sial Dep IIA	84	2.8 (7.5)/0.0 (0.0–0.0)	12 (14.6)
W2mef GIA	84	11.3 (15.6)/6.0 (0.0–19.6)	31 (37.8)
MSP1-19 IIA	84	0.8 (2.7)/0.0 (0.0–0.0)	5 (6.1)
D10 GIA	84	4.2 (8.5)/0.0 (0.0–4.6)	18 (22.0)

a IQR, interquartile range.

Antigens utilized in serologically measured antibodies were selected to reflect the circulating allele frequencies in the population. For example, MSP1 is the most abundant protein found on the merozoite surface and a vaccine candidate. As the merozoite invades the erythrocyte, MSP1 is processed into several fragments, among which the C-terminal 19-kDa fragment remains on the merozoite surface during invasion (63–65). MSP1-19 is composed of 98 highly conserved amino acids, with the exception of residues 1644 (E/Q), 1691(T/K), 1700 (S/N), and 1701 (R/G). Nonsynonymous changes at these positions result in four predominant haplotypes: ETSR (3D7/PNG-MAD20), EKNG (FUP/Uganda-PA), QKNG (FVO/Wellcome), and QTSR (Indo) (66–69). We found that the frequencies of circulating MSP1-19 haplotypes in this region were 44% EKNG (FUP), 39% QKNG (FVO), 8% ETSR (3D7), and 0% QTSR. Therefore, the MSP1-19 FUP, FVO, and 3D7 alleles of the recombinant proteins used should reflect the circulating alleles at the time. Additionally, antibodies to MSP1-19 haplotypes are thought to be broadly cross-reactive (55). The frequency of AMA1 alleles was not measured for this cohort. However, a study conducted in 2000 measured P. falciparum AMA1 haplotype frequencies in nearby Kilifi and found that there were 78 unique haplotypes in the area but that antibodies to AMA1 3D7, AMA1 FVO, and AMA1 HB3 were highly correlated (70). Thus, using the AMA1 3D7 and FVO alleles in the assays should reflect the circulating alleles at the time of this study.

### IgG antibody prevalence in the longitudinal infant cohort plasma.

**FIG 1:**
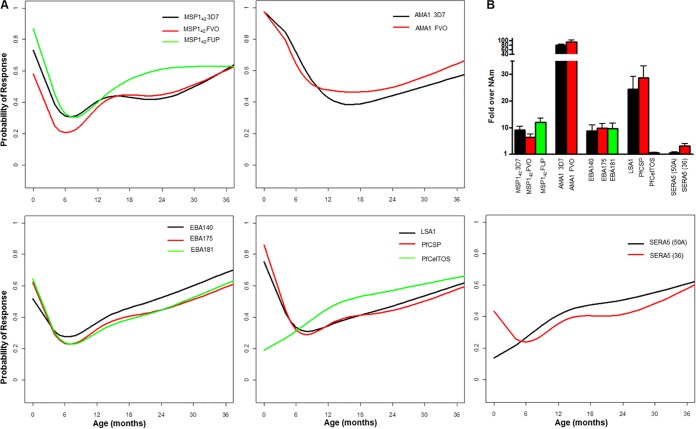
Serological responses in infants over time. (A) Detection probability (*y* axis) of each antibody in infants over time (*x* axis). Responses to specific antigens are indicated in each plot box. (B) Magnitude of antibodies in cord blood for each indicated antigen expressed as fold increase relative to negative-control North Americans (NAm) (mean + standard error of the mean).

Examples of raw data results for the various antibody assays over time are shown in Fig. S1 in the supplemental material. To visualize the complex patterns of antibody responses more clearly, we plotted the probability of the presence of each response over time using restricted cubic splines. Figure 1 illustrates the probability of detecting serologically measured antibody responses over time. In general, maternal antibodies against each recombinant antigen measured in cord blood waned to a nadir by 6 to 9 months of age. The probability of having IgG antibodies to each antigen then increased over time and generally returned to the prevalence observed in cord blood by 36 months of age. Antibodies to AMA1 (3D7 and FVO) were of the highest magnitude in cord blood and did not wane as rapidly as other antibodies. Antibodies to PfCelTOS and SERA5 were essentially absent from cord blood, with infants and young children gradually acquiring IgG antibodies to these antigens over 36 months.

**FIG 2:**
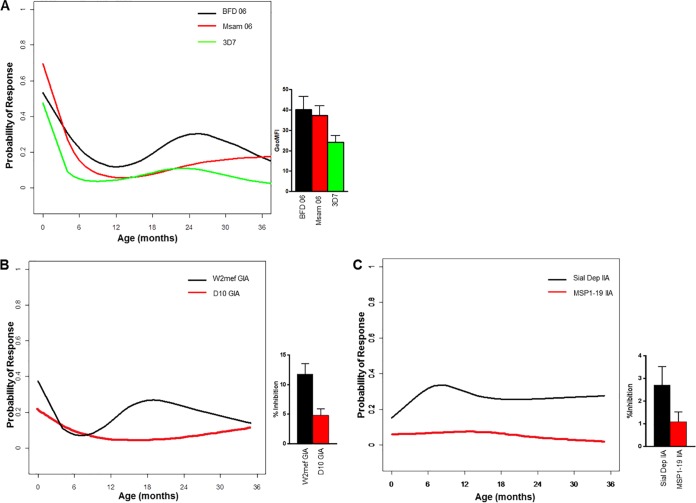
Infant VSA, GIA, and IIA antibodies over time. (A) Detection probability (*y* axis) of each VSA measured antibody response in infants over time (*x* axis). VSA responses to each P. falciparum isolate are indicated. Bar graph to the right of the plot shows the geometric mean fluorescence intensity (GeoMFI) for VSA antibodies in cord blood (mean + standard error of the mean [SEM]). (B) Detection probability (*y* axis) of W2mef and D10 GIA antibody responses in infants over time (*x* axis). Bar graph to the right of the plot shows the percentage of growth inhibition of GIA responses in cord blood (mean + SEM). (C) Detection probability (*y* axis) of Sial Dep and MSP1-19 IIA antibody responses in infants over time (*x* axis). Bar graph to the right of the plot shows the percentage of invasion inhibition responses in cord blood (mean + SEM).

VSA antibodies waned by 6 to 9 months of age and were not (re)acquired during infancy and early childhood (Fig. 2A). W2mef GIA, though not highly prevalent in cord blood, waned by 6 months of age and subsequently (re)appeared at a low rate, while D10 GIA had a consistently negligible prevalence (Fig. 2B). Sial Dep IIA prevalence was overall higher than MSP1-19 IIA, but both were low throughout infancy (Fig. 2C).

We used a generalized estimation equation regression model to estimate the rate of change in the probability of the presence of each antibody response over time using cord blood antibody levels as the baseline or comparator group. Due to the presence of nonlinear relationships in the curves, segmented linear spline terms were used to provide separate estimates of the odds ratios per 1 month change in age within the first 6 months after birth and after 6 months of age. The exception for this analysis was for serologically measured AMA1 antibodies, where we used 12 months of age as the cutoff. The magnitude of cord blood antibodies against AMA1 was high, and infant catabolism of these reached a nadir at 12 months; thus, we compared the rate of waning to the rate of acquisition based on this time point. Additionally, we tested whether there was a significant difference between rates of change of antibody responses before and after 6 months of age. Table 2 presents this analysis for each antibody response as it relates to age ≤6 months and >6 months. As an example, during the first 6 months after birth, there was an associated 25% odds reduction each month in the presence of EBA181 antibodies (odds ratio, 0.75; 95% confidence interval [CI], 0.68 to 0.82; *P* < 0.001), while, after 6 months of age, there was an associated 6% higher odds each month for the presence of EBA181 antibodies (odds ratio, 1.06; 95% CI, 1.03 to 1.08; *P* < 0.001). Similar results were observed for the other serologically measured antibody responses. With regard to VSA, taking the VSA BFD 2006 reactive IgG as an example, during the first 6 months after birth, each month of age was associated with 24% reduced odds of detecting this antibody (odds ratio, 0.76; 95% CI, 0.69 to 0.84; *P* < 0.001), and, after 6 months of age, this odds value did not significantly increase (odds ratio, 1.01; 95% CI, 0.99 to 1.03; *P* = 0.33). Similar results were observed for the other P. falciparum isolates. In general, serologically measured responses waned by 6 to 12 months of age and subsequently increased to reach their highest prevalence by 36 months. In contrast, VSA, GIA, and IIA antibodies waned by 6 to 9 months of age and were not acquired to any great extent during infancy and early childhood. Fig. S2 in the supplemental material illustrates the difference in acquisition/dynamics of serologically measured antibodies versus VSA, GIA, and IIA antibodies. Based on the antibody dynamics observed, a generalized estimation equation regression model was used to assess if the mean rate of change in the probability of having serologic anti-malaria antibodies after 12 months was different than the mean rate of change in the probability of detecting VSA, GIA, and IIA antibodies. Averaging over 13 serological antibody responses, the probability of detecting antibodies increased significantly after 12 months of age. Each month of age was associated with 5% higher odds of detecting the antibodies (odds ratio, 1.05; 95% CI, 1.02 to 1.08; *P* = 0.002). Averaging over the 7 VSA, GIA, and IIA antibody responses, the probability of detecting antibodies did not change significantly over time after 12 months (odds ratio, 0.99; 95% CI, 0.98 to 1.00; *P* = 0.16). The mean rate of change in the probability of detecting 13 serological antimalarial antibody responses after 12 months was significantly different than the mean rate of change in the probability of detecting 7 VSA, GIA, and IIA antibodies after 12 months (*P* < 0.001). Thus, infants acquired serologically measured antibodies but not VSA, GIA, and IIA antibodies after 12 months of age.

**TABLE 2:** IgG antibody rates of change before and after 6 months of age

Assay/antigen tested (P. falciparum isolate)	Time period	Odds ratio (95% CI^*a*^) per 1 mo age change	*P*	*P* difference between rates of change^*b*^
Serology				
LSA1	Before 6 mo^*c*^	0.72 (0.65–0.80)	<0.001	<0.001
	After 6 mo^*d*^	1.04 (1.02–1.07)	<0.001	
CSP	Before 6 mo	0.64 (0.57–0.72)	<0.001	<0.001
	After 6 mo	1.04 (1.02–1.06)	<0.001	
PfCelTOS	Before 6 mo	1.16 (1.04–1.28)	<0.001	0.08
	After 6 mo	1.05 (1.03–1.07)	<0.001	
SE50	Before 6 mo	1.20 (1.07–1.34)	0.0025	0.03
	After 6 mo	1.04 (1.02–1.06)	<0.001	
SE36	Before 6 mo	0.88 (0.80–0.98)	0.01	0.003
	After 6 mo	1.04 (1.03–1.06)	<0.001	
MSP1_42_ (3D7)	Before 6 mo	0.75 (0.68–0.82)	<0.001	<0.001
	After 6 mo	1.04 (1.02–1.06)	<0.001	
MSP1_42_ (FVO)	Before 6 mo	0.79 (0.73–0.87)	<0.001	<0.001
	After 6 mo	1.05 (1.03–1.08)	<0.001	
MSP1_42_ (FUP)	Before 6 mo	0.66 (0.58–0.74)	<0.001	<0.001
	After 6 mo	1.05 (1.03–1.08)	<0.001	
EBA140	Before 6 mo	0.85 (0.77–0.92)	<0.001	<0.001
	After 6 mo	1.06 (1.04–1.09)	<0.001	
EBA175	Before 6 mo	0.76 (0.70–0.84)	<0.001	<0.001
	After 6 mo	1.05 (1.03–1.07)	<0.001	
EBA181	Before 6 mo	0.75 (0.68–0.82)	<0.001	<0.001
	After 6 mo	1.06 (1.03–1.08)	<0.001	
AMA1 (3D7)	Before 12 mo	0.76 (0.71–0.81)	<0.001	<0.001
	After 12 mo	1.03 (1.01–1.06)	0.02	
AMA1 (FVO)	Before 12 mo	0.79 (0.75–0.83)	<0.001	<0.001
	After 12 mo	1.04 (1.01–1.07)	0.003	
Variant surface antigen assay				
BFD 2006	Before 6 mo	0.76 (0.69–0.84)	<0.001	<0.001
	After 6 mo	1.01 (0.99–1.03)	0.33	
Msambweni 2006	Before 6 mo	0.60 (0.53–0.68)	<0.001	<0.001
	After 6 mo	1.02 (0.98–1.05)	0.35	
3D7	Before 6 mo	0.63 (0.55–0.73)	<0.001	<0.001
	After 6 mo	1.01 (0.97–1.05)	0.63	
Growth and invasion inhibition assay				
Sial Dep IIA	Before 6 mo	1.18 (1.04–1.34)	0.01	0.02
	After 6 mo	0.98 (0.93–1.04)	0.56	
W2mef GIA	Before 6 mo	0.77 (0.68–0.89)	<0.001	<0.001
	After 6 mo	1.05 (1.00–1.10)	0.07	
MSP1-19 IIA	Before 12 mo	1.03 (0.94–1.13)	0.53	0.31
	After 12 mo	0.93 (0.81–1.08)	0.35	
D10 GIA	Before 12 mo	0.86 (0.78–0.95)	0.004	0.07
	After 12 mo	1.05 (0.92–1.19)	0.491	

a CI, confidence interval.

b Difference between rates of change between birth to 6 (or 12) months of age and between 6 months of age and 36 months of age (Fisher's exact test).

c Compared to cord blood responses.

d Compared to 6-month responses.

**FIG 3:**
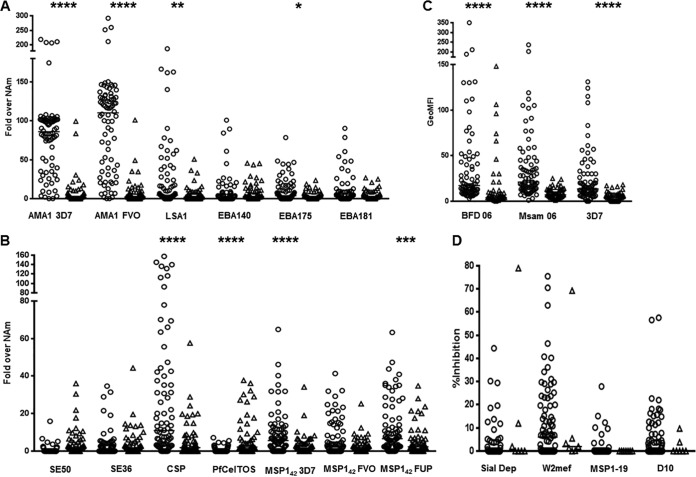
Antibodies in cord blood (open circles) compared to 36-month-old young children (open triangles). Dot plots of serologic antibody responses (fold increase relative to North Americans [NAm]) to specified antigens measured in cord blood and 36-month-old young children (A) and to another set of antigens measured in cord blood and 36-month-old young children (B). Note the smaller *y* axis in (B) than in (A). (C) GeoMFI of VSA responses measured in cord blood and 36-month-old young children. (D) Percent growth inhibition of GIA and IIA antibody responses measured in cord blood and 36-month-old young children. *, *P* = 0.04; **, *P* = 0.0005; ***, *P* = 0.0002; ****, *P* < 0.0001; horizontal bar when visible represents median values.

While the prevalence of serologic antibody responses at 36 months of age was similar to cord blood prevalence, the magnitude of antibody responses was considerably lower. Cord blood levels of antibodies against MSP1_42_ (3D7, FVO, FUP), AMA1 (3D7, FVO), LSA1, EBA175, SE50, and CSP were significantly higher than in 36-month-old young children (Fig. 3A and B). The levels of the 3 VSA antibodies were also significantly higher in cord blood than in 36-month-old young children (Fig. 3C). The GIA and IIA antibodies, however, were low in both groups (Fig. 3D). The only exception to this trend was serologically measured antibodies against PfCelTOS that, in cord blood, had a median of 1, whereas the 36-month-old young children had a median of 3.1-fold increase relative to malaria-naive North American negative controls (*P* < 0.0001, Mann Whitney test; Fig. 3B). It is unknown whether this increase has biological relevance, although it is noted that the magnitude of response is considerably lower than other antigens.

### Fetal sensitization to malaria and acquisition of IgG antibodies.

We hypothesized that newborns that were sensitized to malaria antigens *in utero* would have antibody responses to multiple antigen targets of broader diversity (serologic and functional) at earlier time points during infancy compared to newborns that were not sensitized. In this cohort, 76 infants had complete sensitization and antibody data. Thirty neonates were classified as sensitized, and 46 neonates were classified as not sensitized, based on CBMC recall responses to malaria antigens, as measured by IFN-γ ELISPOT and cytokine production. We found no statistical difference in either the prevalence or magnitude of IgG antibodies in cord blood regardless of sensitized or not sensitized categorization. Examination of longitudinal data revealed no difference in the rate of change (waning or acquisition) for any antimalarial antibodies between sensitized and not sensitized infants/young children over time (Table S1 in the supplemental material). Thus, fetal sensitization to malaria antigens did not affect subsequent infant acquisition of any antimalarial antibody measured in this cohort.

### Infants with serologically measured antimalarial antibody responses were more likely to incur P. falciparum infection.

We examined the association between cord blood and infant antibodies and the risk of P. falciparum infection. The first occurrence of infection was measured by PCR, blood smear, and/or ≥6 positive antimalarial IgM responses to the 13 tested antigens. IgM positivity was used as a marker of recent infection. If an infant had IgM antibodies at one time point, invariably they were absent at the following time point, as has been demonstrated by others (6, 10, 11). Thirty infants had P. falciparum infections detected by 36 months of age. Infections were detected in 7 infants younger than 12 months of age. With respect to the prevalence of maternal antimalarial IgG antibodies in cord blood, there was no difference between infants who incurred malaria infections during the entire follow-up period and those who did not. The paucity of malaria infections in infants younger than 12 months prohibited further analyses regarding sensitization status or characterization of antibody responses.

To exclude confounding maternal antibody responses, we examined the risk of P. falciparum infection after 12 months of age as related to infant antibody responses at the 12-month time point. Sixty-seven infants had 12-month antibody data. Within this subset, malaria infections were detected in 17 infants in the subsequent 24 months of follow-up. Using a Cox proportional hazards regression model, we found that infants with serologically measured antibodies at 12 months were more likely to incur malaria infections than infants who were seronegative (Table 3). Specifically, 12-month-old infants who had IgG antibodies to CSP, SERA5, MSP1_42_ (FVO), MSP1_42_ (FUP), EBA140, EBA175, AMA1 (3D7), or AMA1 (FVO) had a statistically significant increased risk of infection (hazard ratio range, 2.64 to 6.21) compared to infants with negative serology at 12 months of age. No increased risk was associated with VSA, GIA, and/or IIA antibody at 12 months, though the prevalence of these antibodies was low.

**TABLE 3:** Association between presence of IgG antibodies at 12 months of age and first occurrence of a malaria infection after 12 months of age

Assay/antigen tested	No. tested	No. of malaria events	Hazard ratio (95% confidence interval)	*P*
Serology				
LSA1	67	17	1.84 (0.71–4.77)	0.21
CSP	67	17	2.64 (1.00–6.97)	0.05
PfCelTOS	67	17	2.67 (0.94–7.61)	0.07
SE50	67	17	4.51 (1.46–13.9)	0.009
SE36	67	17	2.35 (0.89–6.19)	0.08
MSP1_42_ (3D7)	67	17	1.90 (0.73–4.99)	0.19
MSP1_42_ (FVO)	67	17	4.37 (1.52–12.6)	0.006
MSP1_42_ (FUP)	67	17	3.13 (1.14–8.57)	0.03
EBA140	67	17	3.27 (1.19–8.95)	0.02
EBA175	67	17	3.12 (1.18–8.25)	0.02
EBA181	67	17	1.76 (0.68–4.56)	0.25
AMA1 (3D7)	67	17	2.98 (1.07–8.33)	0.04
AMA1 (FVO)	67	17	6.21 (1.67–23.1)	0.006
Variant surface antigen assay				
BFD 2006	67	17	2.43 (0.78–7.56)	0.13
Msambweni 2006	67	17	1.00 (0.13–7.60)	1.00
3D7	67	17	2.45 (0.56–10.8)	0.23
Growth and invasion inhibition assay				
Sial Dep IIA	65	17	1.64 (0.60–4.47)	0.34
W2mef GIA	65	17	1.52 (0.49–4.74)	0.47
MSP1-19 IIA	65	17	1.56 (0.36–6.84)	0.55
D10 GIA	65	17	1.49 (0.20–11.3)	0.70

## DISCUSSION

Early infancy is a critical time in the development of immunity to malaria in children born in areas of malaria endemicity. Whereas a relative degree of protection from P. falciparum infection and symptomatic malaria is thought to exist from birth to approximately 6 months of age (4–6, 71–73), subsequent exposure to mosquito-borne transmission during early infancy is accompanied by the absence of *de novo* synthesized fetal hemoglobin and the catabolic loss of maternal IgG antibodies that have passed from the maternal to fetal circulation during the last trimester of pregnancy. More generally, the infant immune system is immature until at least 2 years of age (74). Our prospective study of infants born in an area of malaria endemicity of coastal Kenya from 2006 to 2009 was performed to understand in more detail the interplay between the loss of malaria antigen-specific maternal malarial antibodies present at birth and the subsequent acquisition of infant antibodies that result from natural exposure to P. falciparum. In addition to measuring serologic IgG antibodies to P. falciparum antigens from birth through early infancy, as several other studies have reported (6, 12, 75), we performed several assays that reflect functional antibody responses that include antibody binding to VSAs expressed on the surface of P. falciparum-infected erythrocytes, GIA, and IIA specific for MSP1, and sialic acid-dependent erythrocyte invasion by merozoites. The main conclusions from our study indicate that (i) serologic measures of maternal IgG antibodies to preerythrocytic and blood stage antigens wane by 6 months after birth and reappear over the following 24 to 36 months as a consequence of natural malaria exposure; (ii) increased levels of serologic antibodies at 12 months of age are predictive of an increased subsequent risk of P. falciparum infection; (iii) VSA and functional antibody responses mediated by maternal antibodies in cord blood disappear within 6 months after birth and, unlike serologically determined antibodies, remain low up to 36 months of age; (iv) *in utero* sensitization to P. falciparum is not associated significantly with enhanced antibody responses following the loss of maternal IgG antibodies.

Our observations related to serologic maternal malaria IgG antibodies present at birth (Table 1) indicate that antigenic targets of such antibodies are expressed by both preerythrocytic (CSP, LSA1, PfCeltos) and blood stage (e.g., MSP1, EBA140, EBA175, EBA181, AMA1, SERA5) parasites. These antibodies decreased significantly by 6 months after birth and then gradually increased up to age 36 months. Maternal IgG antibodies detectable in cord blood have previously been reported to be directed against ring-infected erythrocyte surface antigen (RESA), CSP, MSP1-19, MSP3, AMA1, EBA175, and glutamate-rich protein (GLURP) (6, 11-13, 75). Studies of other birth cohorts in sub-Saharan Africa have reported the waning of maternal malarial IgG antibodies by 6 to 9 months of age. Following this loss of maternal antibodies, the level of serologically detectable malaria-specific IgG antibodies gradually increased up to 36 months (Fig. 1). However, these newly acquired antibodies were associated not with protective immunity but with an increased prospective risk of infection, most likely due to increased exposure. Elevated levels of malaria IgG antibodies has previously been found to be a biomarker of increased malaria risk during early infancy in a birth cohort study from Ghana (6). Although we did not compare antibody levels in young infants with those of older children in the Kenyan study population described here, a recent study by Stanisic et al. (14) did so in cohorts of 1- to 4- and 5- to 14-year-old Papua New Guinean children. Results of this study in Papau New Guinea indicate that one of the reasons why antibody responses in young infants represent biomarkers of malaria exposure rather than protection from malaria is related to failure of antibody responses to reach a critical protective level, as determined by serology, until age 4 years or older. Mathematical models of antibody half-lives in cohorts of younger and older African children suggest that antibodies in younger children have shorter half-lives than those of older children and that this difference in half-lives may be related to differing populations of long-lived and short-lived antibody-secreting cells in the two age groups (76). Although not measured in this study, IgG subclasses may affect the longevity of circulating antibodies. In general, malaria infection predominantly induces IgG1 and IgG3 isotypes to various P. falciparum-specific antigens (14, 52, 77–81). In contrast to serologic measures of antibody responses, our data indicate that functional assays of antibody activity are overall weak at birth, decrease by 6 months, and do not reappear by 36 months of age (Fig. 2). We have previously shown in different infants examined from the same cohort that GIA (D10, 3D7, W2mef, Msam 06) decreased in infants over time until 12 months of age (30). In the present study, only W2mef GIA increased transiently at 18 months of age, but it had low prevalence by 36 months of age. This indicates that, if boosted, the resultant antibodies were short-lived in these 18-month-old young children. It may be that in this infant cohort, antibodies to merozoite antigens did not reach sufficiently high levels to mediate substantial invasion-inhibitory activity. Prior studies have suggested that GIA antibodies are not readily boosted by increasing exposure to malaria (15, 27). However, in young children, GIA antibodies showed some association with malaria exposure transmission level (27). VSA antibodies were moderately prevalent (47% to 69%) in cord blood, indicating that mothers who were children themselves during a time of higher malaria transmission in coastal Kenya (34) had VSA antibodies transferred to their fetuses in the third trimester. This infant cohort with lower malaria exposure, on the other hand, failed to develop much VSA antibody. Of note, a trend toward higher prevalence of VSA antibodies to BFD06, a parasite taken from a patient with severe malaria, was noted in infants approximately 24 months of age. Others have proposed that children develop VSA antibodies to parasites expressing VSA associated with severe disease earlier in childhood than to those expressing VSA associated with mild or moderate disease (82). These findings are similar to those of Vestergaard et al. (21), who showed that infants residing in a low malaria transmission region of Tanzania had low prevalence and magnitude of VSA antibodies compared to infants residing in regions of high transmission. Nhabomba et al. (11) also found a lack of VSA antibody acquisition in infants up to 2 years of age in Mozambique. Conversely, the parasite isolates used in our study may not have been an accurate representation of the circulating parasites from the region, despite one isolate coming from a child with nonsevere acute malaria from this study cohort. Antibodies to VSA are known to be highly isolate specific among children (83, 84), and may be short lived (85). Therefore, the prevalence of antibodies to any one isolate may be low in young children, as we found here. With respect to antibodies that function to impair merozoite invasion of erythrocytes, we used two assays that assess antibodies to the 19-kDa C-terminal region of MSP1 and antibodies that target sialic acid-dependent invasion pathways (16, 32). MSP1 is involved with the initial low affinity binding of the merozoite to the erythrocyte, with the MSP1-19 portion of the cleaved MSP1 retained as the merozoite invades (86). A secondary interaction is then required with ligands of the EBA family and P. falciparum reticulocyte-binding homolog (PfRh) proteins (87). The variable expression of these proteins facilitates the merozoite invading through roughly grouped phenotypes labeled sialic acid-dependent or sialic acid-independent, and variation in their use facilitates evasion of acquired antibodies (32). W2mef generally invades through a sialic acid-dependent pathway. When EBA175 is genetically deleted (W2mefΔEBA175), it invades through a sialic acid-independent pathway. Thus, plasma that contains antibodies that bind to EBA175, and other ligands of sialic acid-dependent invasion, may inhibit the invasion of W2mef but not W2mefΔEBA175 parasites. EBA140, EBA181, and PfRh1 may also participate in the sialic acid-dependent pathway (32). With respect to both MSP1-19 and sialic acid-dependent IIA, we found that antibodies with these activities were low to negligible at birth and were not detectable at 36 months. These findings are discordant with serology, as both MSP1_42_ and EBA175 antibodies were detectable at birth and progressively increased in infants between age 12 and 36 months. This could be explained by antibodies to merozoite antigens being at levels below a threshold concentration to effectively inhibit invasion or by antibodies targeting nonfunctional epitopes. This discordance between serologic and functional measures of antibody responses has been described in other studies (31, 88) and highlights the challenge of validating *in vitro* assays relevant to malaria pathogenesis *in vivo*. Recent studies have identified several such potential functional assays that include evaluating antibodies that opsonize merozoites for phagocyotosis or fix complement to inhibit invasion and lyse merozoites (89, 90). Additionally, competitive ELISAs for EBA175 (91) and refined assays examining AMA1 complex responses (92) are in development.

With respect to individual covariates that might impact the acquisition of antibody responses by young infants, we examined the relationship between *in utero* sensitization to malaria and postnatal serologic and functional antibody responses. Studies we conducted in this area of coastal Kenya from 2000 to 2003 have shown that 45% to 60% of newborns were sensitized *in utero* as determined by the cord blood T-cell cytokine responses to malaria antigens (35). This sensitization was associated with rapid acquisition of MSP1-19 IIA relative to newborns that were not sensitized (88). However, in the 2006 to 2009 birth cohort reported here, we found no association of *in utero* T-cell sensitization and accelerated development of antibody responses in young infants. We speculate that this discrepancy is likely related to changes in malaria exposure during the two different time periods. While transmission was stable and relatively high from 2000 to 2003, it decreased significantly from 2007 to 2009 as a result of increasing use of insecticidal bed nets and other public health interventions (34).

In conclusion, results of our study highlight several issues pertinent to the development of naturally acquired immunity during the first 3 years after birth. First, while serologic measures of malaria antigen-specific antibodies are clearly indicative of exposure to P. falciparum, they are unlikely to be relevant to protective immunity as opposed to malaria exposure in young infants. In this context, a limitation of our study is that we did not perform active surveillance for symptomatic malaria; thus, our results can only be linked with susceptibility to P. falciparum infection. Second, results of various birth cohort studies may vary according to prevailing levels of malaria endemicity during gestation, e.g., maternal malaria exposure and maternal antibodies transferred to the fetus, as well as malaria exposure experienced by infants after the waning of maternal antibodies. Third, our results underscore the need for additional functional antibody assays, e.g., phagocytosis of antibody opsonized merozoites and complement fixation, that have been found to correlate with protection from symptomatic malaria (89, 90). This will be challenging, given the likely complexity and redundancy of host immune and nonimmune mechanisms underlying naturally acquired immunity to malaria.

## Supplementary Material

Supplemental material
