# Supplementary material for: Contrasting Patterns of Serologic and Functional Antibody Dynamics to Plasmodium falciparum Antigens in a Kenyan Birth Cohort
Source: Clin Vaccine Immunol. 2016 Feb 5;23(2):104–16. doi: 10.1128/CVI.00452-15 (PMC4744923; doi:10.1128/CVI.00452-15)
Supplement: Supplemental material [file supp_23_2_104__index.html]

Contrasting Patterns of Serologic and Functional Antibody Dynamics to Plasmodium falciparum Antigens in a Kenyan Birth Cohort — Supplemental material 

# Contrasting Patterns of Serologic and Functional Antibody Dynamics to Plasmodium falciparum Antigens in a Kenyan Birth Cohort

## Supplemental material

- Supplemental file 1 -

  Table S1. IgG antibody rates of change before and after 6 months of age in malaria-sensitized versus -not sensitized infants. Fig. S1. Three examples of the raw data for the longitudinal infant cohort. Fig. S2. Detection probability of infant antibodies over time.

  PDF, 214K
