# Supplementary material for: Contrasting Patterns of Serologic and Functional Antibody Dynamics to Plasmodium falciparum Antigens in a Kenyan Birth Cohort
Source: Clin Vaccine Immunol. 2016 Feb 5;23(2):104–16. doi: 10.1128/CVI.00452-15 (PMC4744923; doi:10.1128/CVI.00452-15)
Supplement: Supplemental material [file CVI.00452-15_zcd999095303so1.pdf]

Supplemental Table 1: IgG antibody rates of change before and after 6 months of age in malaria sensitized vs. not sensitized infants.

**Neonatal Malaria Sensitization Status**		**Not Sensitized**		**Sensitized**		
**Prevalence of Assay/Antigen tested/Pf Isolate**		**Odds Ratio (95%CI) / 1 month**	**P [a]**	**Odds Ratio(95%CI) / 1 month**	**P [a]**	**P difference [b]**
**Serology (fold increase in MFI over negative controls) n=71 infants**						
LSA1	Before 6 months [c]	0.69 (0.59,0.82)	< 0.001	0.71 (0.62,0.82)	< 0.001	0.82
	After 6 months [d]	1.05 (1.02,1.09)	0.001	1.04 (1.00,1.07)	0.03	0.46
CSP	Before 6 months	0.67 (0.58,0.79)	< 0.001	0.61 (0.48,0.76)	< 0.001	0.46
	After 6 months	1.03 (1.00,1.06)	0.03	1.05 (1.01,1.09)	0.02	0.56
PfCelTOS	Before 6 months	1.11 (0.95,1.29)	0.19	1.24 (1.07,1.45)	0.005	0.30
	After 6 months	1.06 (1.03,1.09)	< 0.001	1.03 (1.00,1.07)	0.04	0.23
SE50	Before 6 months	1.27 (1.06,1.53)	0.010	1.13 (0.97,1.33)	0.11	0.35
	After 6 months	1.05 (1.02,1.08)	0.001	1.04 (1.01,1.07)	0.02	0.57
SE36	Before 6 months	0.91 (0.79,1.04)	0.15	0.90 (0.77,1.06)	0.20	0.97
	After 6 months	1.05 (1.02,1.07)	< 0.001	1.04 (1.01,1.07)	0.009	0.84
$MSP1_{42}$ (3D7)	Before 6 months	0.80 (0.70,0.92)	0.002	0.71 (0.60,0.82)	< 0.001	0.21
	After 6 months	1.03 (1.00,1.07)	0.05	1.06 (1.02,1.09)	< 0.001	0.30
$MSP1_{42}$ (FVO)	Before 6 months	0.82 (0.72,0.93)	0.002	0.75 (0.65,0.87)	< 0.001	0.38
	After 6 months	1.06 (1.03,1.09)	< 0.001	1.06 (1.02,1.10)	0.002	0.89
$MSP1_{42}$ (FUP)	Before 6 months	0.63 (0.52,0.77)	< 0.001	0.71 (0.60,0.84)	< 0.001	0.39
	After 6 months	1.07 (1.03,1.11)	< 0.001	1.03 (1.01,1.06)	0.02	0.12

Neonatal Malaria Sensitization Status		Not Sensitized		Sensitized		
Prevalence of Assay/Antigen tested/Pf Isolate		Odds Ratio (95%CI) / 1 month	P [a]	Odds Ratio(95%CI) / 1 month	P [a]	P difference [b]
EBA140	Before 6 months	0.82 (0.72,0.93)	0.002	0.88 (0.78,1.01)	0.06	0.42
	After 6 months	1.08 (1.05,1.12)	< 0.001	1.04 (1.00,1.08)	0.03	0.12
EBA175	Before 6 months	0.79 (0.70,0.90)	< 0.001	0.69 (0.59,0.82)	< 0.001	0.21
	After 6 months	1.05 (1.02,1.08)	< 0.001	1.06 (1.02,1.10)	0.001	0.76
EBA181	Before 6 months	0.82 (0.73,0.92)	0.001	0.64 (0.53,0.78)	< 0.001	0.03
	After 6 months	1.05 (1.02,1.08)	0.001	1.06 (1.02,1.11)	0.003	0.60
AMA1 (3D7)	Before 12 month	0.78 (0.71,0.85)	< 0.001	0.76 (0.69,0.84)	< 0.001	0.79
	After 12 months	1.04 (1.00,1.07)	0.06	1.02 (0.98,1.08)	0.33	0.73
AMA1 (FVO)	Before 12 month	0.81 (0.76,0.87)	< 0.001	0.77 (0.70,0.85)	< 0.001	0.38
	After 12 months	1.04 (1.00,1.08)	0.04	1.04 (0.99,1.09)	0.11	0.94
**Variant Surface Antigen Assay (Geometric mean MFI) n=72 infants**						
BFD 2006	Before 6 months	0.80 (0.71,0.91)	< 0.001	0.66 (0.54,0.81)	< 0.001	0.10
	After 6 months	1.03 (1.00,1.05)	0.06	1.00 (0.95,1.04)	0.93	0.30
Msambweni 2006	Before 6 months	0.61 (0.53,0.70)	< 0.001	0.58 (0.46,0.72)	< 0.001	0.71
	After 6 months	1.02 (0.97,1.06)	0.47	1.02 (0.96,1.08)	0.48	0.87
3D7	Before 6 months	0.62 (0.52,0.74)	< 0.001	0.65 (0.51,0.84)	0.001	0.75
	After 6 months	1.02 (0.96,1.08)	0.58	1.00 (0.94,1.07)	0.92	0.75
**Growth and Invasion Inhibition Assays (Percent Inhibition) n=76 infants**						

Neonatal Malaria Sensitization Status		Not Sensitized		Sensitized		
Prevalence of Assay/Antigen tested/Pf Isolate		Odds Ratio (95%CI) / 1 month	P [a]	Odds Ratio(95%CI) / 1 month	P [a]	P difference [b]
Sialic Acid Dep IIA	Before 6 months	1.15 (0.97,1.37)	0.10	1.21 (0.96,1.53)	0.10	0.74
	After 6 months	1.01 (0.95,1.08)	0.76	0.97 (0.89,1.05)	0.39	0.39
W2mef GIA	Before 6 months	0.74 (0.62,0.90)	0.002	0.82 (0.65,1.03)	0.09	0.53
	After 6 months	1.06 (1.00,1.13)	0.06	1.00 (0.92,1.10)	0.93	0.32
MSP1-19 IIA	Only 2 positive responses in malaria sensitized infants and 0 positive responses in not sensitized infants.					
D10 GIA	Over entire follow up period	0.93 (0.85,1.02)	0.12	0.77 (0.60,1.00)	0.05	0.17

[a] Difference between rates of change between birth to 6 (or 12) months of age and between 6 months of age and 36 months of age (Fisher's exact test). [b] Difference between rates of change in sensitized vs. not sensitized infants. [c] Compared to cord blood responses. [d] Compared to 6 month responses.

Supplementary Figure Legends

SFigure 1. Three examples of the raw data for the longitudinal infant cohort. The top panel shows serology for $MSP1_{42}$ (FUP) results, the middle panel shows Msam 06 VSA results, and the bottom panel shows W2mef GIA results measured in infants over time (x axis).

SFigure 2. Detection probability of infant antibodies over time. Detection probability (y axis) of all measured antibody responses in infants over time (x axis, months). Serologic responses are shown with solid colored lines. VSA, GIA, and IIA responses are shown with dashed colored lines.

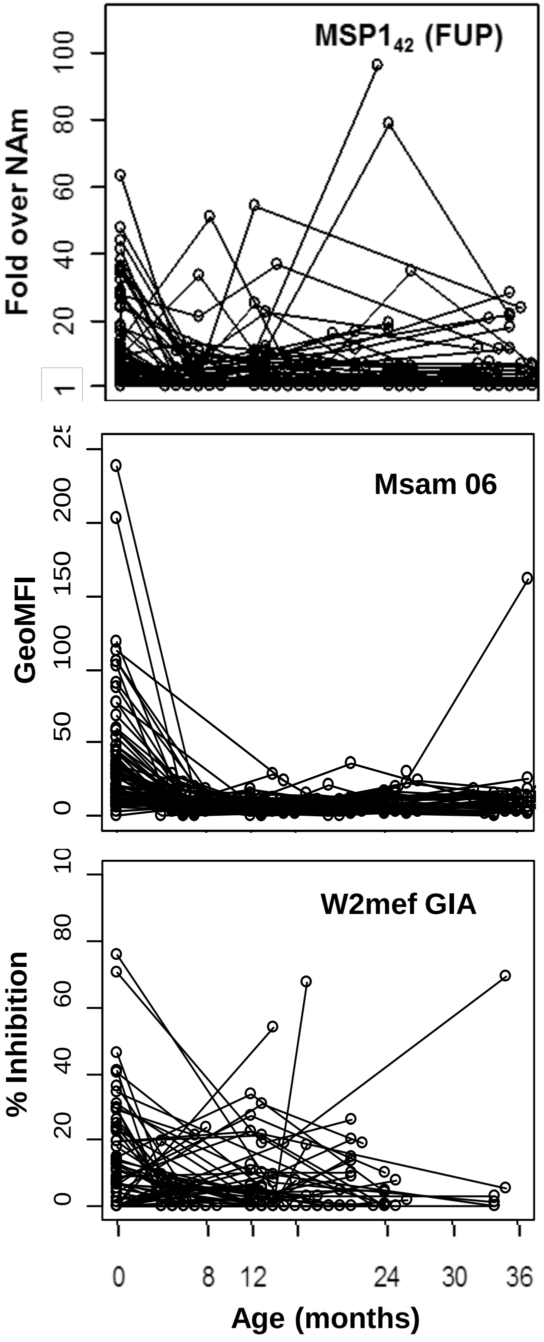

Supplemental Figure 1

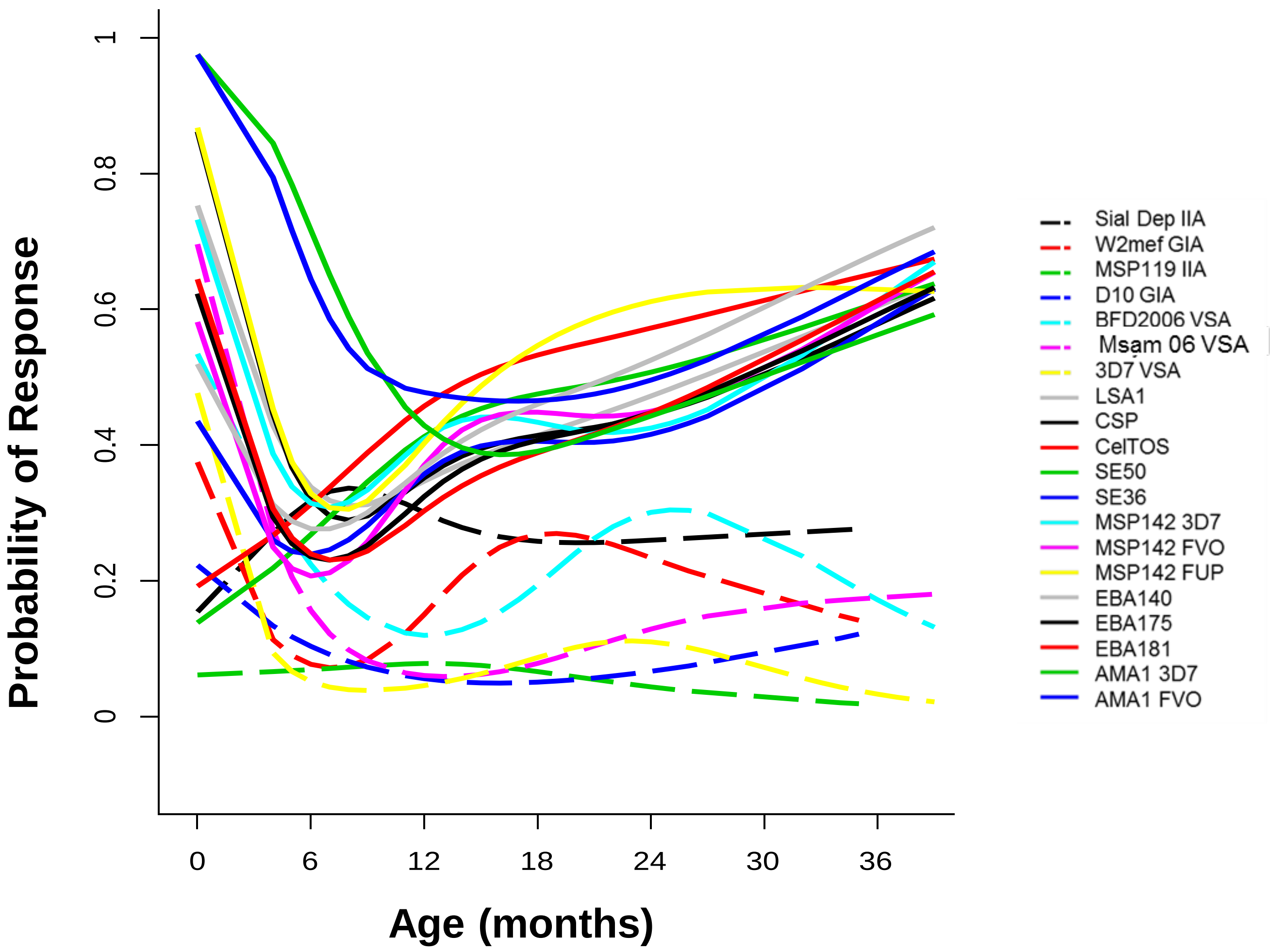

Supplemental Figure 2
